# Strangulated Tension Viscerothorax with Gangrene of the Stomach in Missed Traumatic Diaphragmatic Rupture

**DOI:** 10.5402/2011/458390

**Published:** 2011-06-22

**Authors:** Uvie Onakpoya, Akinwumi Ogunrombi, Anthony Adenekan, William Akerele

**Affiliations:** ^1^Cardiothoracic Surgery Unit, Department of Surgery, College of Health Sciences, Obafemi Awolowo University, Ile-Ife 220005, Nigeria; ^2^Department of Anaesthesia, College of Health Sciences, Obafemi Awolowo University, Ile-Ife 220005, Nigeria

## Abstract

Acquired diaphragmatic hernias are usually posttraumatic in occurrence. In patients who have blunt trauma and associated diaphragmatic hernia, the diagnosis may be missed or delayed, often leading to poor treatment outcomes. We present a rare occurrence of tension viscerothorax due to missed traumatic diaphragmatic rupture in a 25-year-old woman whose condition was complicated by gangrene and perforation of the fundus as well as questionable viability of the anterior wall of the body of the stomach. The patient had a successful emergency transabdominal suture plication of the diaphragm and gastroplasty and has remained symptomless 3 months postoperatively.

## 1. Introduction

Acquired diaphragmatic hernias are usually posttraumatic in occurrence. They occur following motor vehicular accidents, falls, and stabs or after laparoscopic upper abdominal surgeries [1]. Though penetrating chest and abdominal injuries have higher chances of causing diaphragmatic hernias, it is well known that blunt trauma is associated with the condition. The diagnosis of a diaphragmatic rent is often made in patients who suffer penetrating abdominal injuries because they have higher incidences of operative intervention and as such, the diagnosis is usually made intraoperatively. However, in patients who have diaphragmatic hernia due to blunt trauma, the diagnosis may be missed or delayed, leading to poor outcomes [2, 3]. Tension viscerothorax occurring as a result of a traumatic diaphragmatic hernia is very rare [4]. 

We present a case of tension viscerothorax occurring in patient with missed traumatic diaphragmatic rupture whose condition was complicated by gangrene and perforation of the fundus and questionable viability of the anterior wall of the body of the stomach.

## 2. Case Report

A 25-year-old lady was referred from a medical facility in 2010 where she had previously been managed for 8 weeks for a left shoulder avulsion injury and blunt chest trauma sustained during a motor vehicular accident. She had a successful skin grafting and was considered for discharge home a day before she was referred to us when she suddenly developed difficulty with breathing, dull aching central chest pain, and palpitations. She was referred on intravenous dopamine support due to a cardiovascular collapse and was immediately admitted into the intensive care unit (ICU) of the Obafemi Awolowo University Teaching Hospital. 

**Figure 1:**
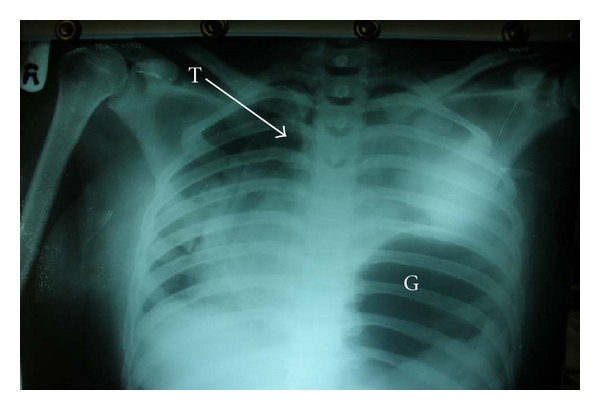
Preoperative mobile chest X-ray showing a large gastric shadow (G), absence of the left diaphragmatic outline and rightward displacement of the cardiac silhouette and trachea (T).

**Figure 2:**
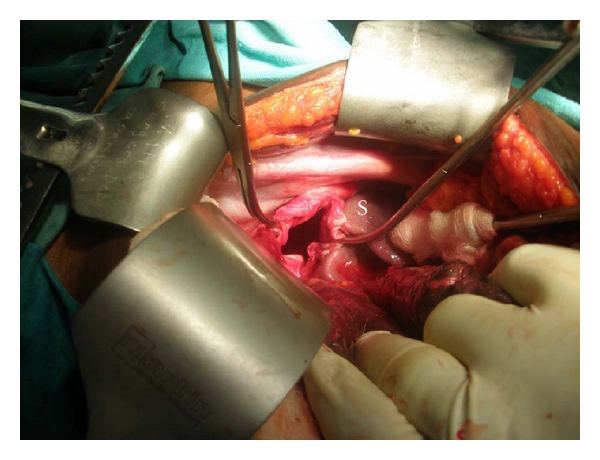
Intra-operative picture showing the diaphragmatic defect after reduction of the stomach. Shown here is the spleen (S). The gastric fundus is retracted by the gloved hand.

**Figure 3:**
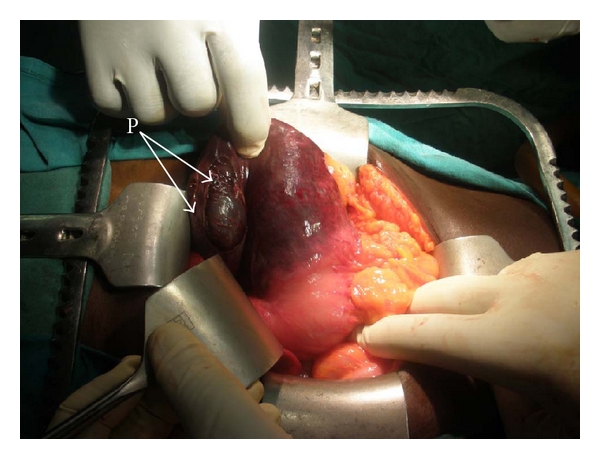
Intra-operative picture demonstrating the sharp demarcation between the viable portions of the stomach and areas of questionable viability. Also seen is the area on the fundus with sloughed off seromuscular layers and pouting mucosa with two small perforations (P).

**Figure 4:**
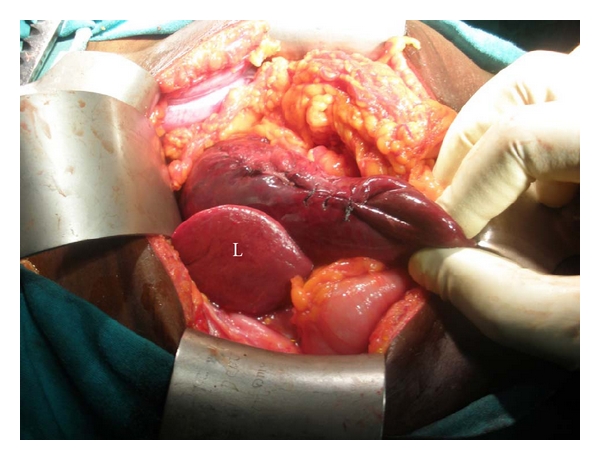
Completed gastroplasty. The liver (L) is shown to the right of the stomach.

**Figure 5:**
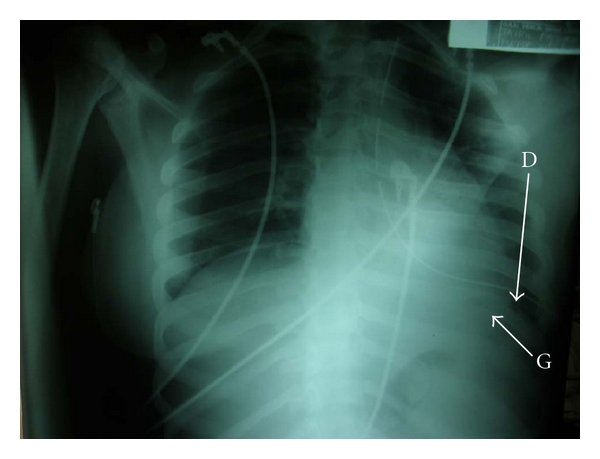
Immediate post-operative chest X-ray demonstrating a well formed left diaphragmatic outline (D), cardiac silhouette and an intra-abdominal gastric gas shadow (G).

Examination revealed a profusely sweaty young woman who was pale, dehydrated, and in severe respiratory distress (respiratory rate, 58 cycles/min) despite being on intranasal oxygen at 9 Litres/minute, hypotensive (unrecordable blood pressure) and tachycardic (heart rate, 180/min). The heart sounds were heard on the right of the sternum. The trachea was deviated to the right and there was reduced chest movement and reduction of the vocal resonance on the left hemithorax. The abdominal examination was unremarkable while a dry dressing was applied over the left shoulder region and left upper arm. An urgent mobile chest X-ray (Figure 1) showed a loss of left diaphragmatic outline and a large gastric shadow occupying the left hemithorax and displacing the mediastinum to the right. A diagnosis of a missed left-sided traumatic diaphragmatic rupture with tension gastrothorax was made. A nasogastric tube was inserted without difficulty but with no appreciable improvement in her clinical condition. She was administered broad-spectrum antibiotics (Intravenous Cefuroxime 1.5 g stat and Metronidazole 500 mg stat) as well as intravenous fluids (Isotonic saline 130 mLs/hour). Acceptable tissue perfusion was ensured with a urinary output of 35 mLs/hour (0.7 mL/kg/hour), and she was expeditiously taken to the operating room 2 hours after arrival at the ICU. An upper midline laparotomy incision was used to access the abdominal cavity where an 8 cm rent was noticed in the left dome of the diaphragm (Figure 2) as well as the herniated stomach, greater omentum, and spleen. After reduction of the abdominal viscera, about 1000 mL of dark blood-stained fluid was observed in the left hemithorax as well as gangrene of the fundus (with two small perforations) and areas on the anterior wall of the stomach of questionable viability (Figure 3) even after administering 100% oxygen and hot abdominal towels. However, since there was a pulsatile right gastroepiploic artery, a gastroplasty was done by invaginating the involved gangrenous and suspicious areas with seromuscular Lambert sutures of silk 2/0 applied on adjoining viable tissues (Figure 4) with the intention of having a second-look laparotomy 48–72 hours later to assess the stomach. The rationale for a gastroplasty as opposed to a gastric resection of the gangrenous fundal region was to reduce the operative time in this very ill patient. A left closed thoracostomy tube was inserted and the diaphragmatic defect was closed in two layers using nonabsorbable nylon sutures: a first layer of interrupted and a second layer of continuous over—and-over sutures. She made an uneventful postoperative recovery and an immediate post-operative chest X-ray showed an intra-abdominal relocation of the stomach and a well-defined diaphragmatic outline (Figure 5). Since her clinical condition improved dramatically with no undue abdominal tenderness or distension 72 hours post-operatively, it was decided not to reexplore her abdomen. She commenced oral intake on the 5th post-operative day and was discharged home 5 days later. She was last seen in the outpatient's clinic three months after surgery in 2010 and she remains asymptomatic.

## 3. Discussion

That the diagnosis of diaphragmatic rupture particularly following blunt trauma is often delayed has been established by several authors [2, 5]; indeed, chest radiographs often miss up to half of penetrating diaphragmatic ruptures presenting to the emergency department [6]. This therefore necessitates a high index of suspicion in any patient who has sustained a chest or abdominal trauma since there are no pathognomonic features of this condition. Undiagnosed cases usually only present when there is herniation (usually of abdominal viscera) through the diaphragmatic defect causing a “space-occupying lesion” in the thoracic cavity. This occurs due to the differential pressures between the abdominal and thoracic cavities with the positive intra-abdominal pressures and negative intra-thoracic pressures encouraging the movement of abdominal viscera into the thoracic cavity. These “neo-” thoracic viscera then exert pressure due to their size and contents on the lung, heart, and great vessels. Tension viscerothorax causes kinking of the vena cavae and results in a reduction in venous return to the heart and diminished cardiac output [7]; features which because they involve gas-containing organs like the stomach or colon are indistinguishable from tension pneumothorax [4, 8, 9]. Of the various organs that have been described that herniate through the diaphragmatic rupture [10, 11], the stomach is the most common organ due to its proximity to the relatively unprotected left dome of the diaphragm [12, 13]. 

Surgical access to the site of the rupture on the diaphragm is usually dictated by the time interval between injury and diagnosis as well as the suspicion of the intra-abdominal or thoracic visceral injury; as such, the abdominal route is the norm in acute trauma where there may be associated intra-abdominal visceral injury while in chronic situations (usually more than 6 weeks); a thoracotomy is usually performed because it provides a much easier access to divide adhesions between the involved abdominal viscera and the lungs as well as an eminent route for repair of the diaphragmatic defect [13, 14]. The use of laparotomy for delayed presentation of traumatic diaphragmatic rupture was practised by Hegarty and colleagues [15] who were successful in about 50% of cases in reducing the hernia in their patients. They concluded that it was better to approach late defects through the chest because of better success rates of hernia reduction. We elected however in this patient to utilise an upper midline laparotomy because of her poor clinical condition (severe tachycardia and hypotension). It was felt that she would better tolerate a laparotomy and that with expeditious decompression of the left thoracic cavity, her clinical condition would improve. The ease with which this was carried out intra-operatively was enhanced by the relatively minimal adhesions to the left lung and parietal pleura. Fortuitously, this decision enabled us to fully assess the stomach and carry out a gastroplasty, a procedure that would have been a bit more difficult had the patient had a thoracotomy, because the areas of questionable viability on the anterior gastric wall extended to the junction of the gastric body and the antrum (Figure 3). The gastroplasty involved the use of interrupted nonabsorbable silk Lambert sutures applied to apparently healthy adjoining gastric seromuscular layers to invaginate the necrotic gastric fundal region. A resection of the involved necrotic gastric wall could also have been done especially if there was a clear demarcation between viable and nonviable tissues. In this patient however, a gastroplasty was performed because its ease and speed significantly reduced the operative time in this haemodynamically unstable patient. A proximal partial gastrectomy and a total gastrectomy with a Roux-en-Y oesophagojejunostomy are other surgical options that may be indicated in patients with extensive gastric gangrene; but because they are more extensive procedures, they may be associated with increased morbidities and mortalities. Indeed, the surgical outcome following a tension viscerothorax has been generally poor especially when complicated by gangrene and perforation of the stomach as reported by various workers [1, 13, 15]. It is our opinion that the less invasive access of laparotomy over a thoracotomy could have contributed to the favourable outcome in this patient who had cardiopulmonary collapse on presentation.

In conclusion, tension viscerothorax should therefore be considered in patients who have sustained blunt chest and abdominal injury and have features suggestive of a tension pneumothorax. Careful interpretation of chest radiographs, thoughtful consideration in deciding the access to the diaphragmatic repair as well as expeditious resuscitation, and surgical intervention are likely to lead to a good outcome.
